# Long noncoding RNA XIST promotes cell proliferation and migration in diabetic foot ulcers through the miR-126-3p/EGFR axis

**DOI:** 10.1186/s13098-024-01260-9

**Published:** 2024-02-06

**Authors:** Wangbing Hong, Zhenfang Xiong, Xin Wang, Xincheng Liao, Mingzhuo Liu, Zhengying Jiang, Dinghong Min, Jiaqi Li, Guanghua Guo, Zhonghua Fu

**Affiliations:** 1 Medical Center of Burn plastic and wound repair, The 1st Affiliated Hospital, Jiangxi Medical College, Nanchang University, Nanchang, Jiangxi Province China; 2 Department of Pathology, The 1 st Affiliated Hospital, Jiangxi Medical College, Nanchang University, Nanchang, Jiangxi Province China

**Keywords:** Diabetic foot ulcer, LncRNA XIST, miR-126-3p, EGFR

## Abstract

**Background:**

The prevalence of diabetic foot ulcers (DFUs) has caused serious harm to human health. To date, a highly effective treatment is lacking. Long noncoding RNA X-inactive specific transcript (lncRNA XIST) has been the subject of mounting research studies, all of which have found that it serves as a protective factor against certain diseases; however, its function in DFUs is not entirely understood. This study was performed to determine the importance of the lncRNA XIST in the pathogenesis and biological function of DFUs.

**Methods:**

Diabetic ulcer skin from rats was analysed using haematoxylin-eosin (HE), Masson’s trichrome, and immunohistochemistry (IHC) staining. The differences in the expression of genes and proteins were examined with real-time quantitative polymerase chain reaction (RT–qPCR) and Western blotting. Next, the interaction was verified with a dual luciferase gene reporter assay. In addition, CCK-8, Transwell, and wound healing assays were used to assess the proliferation and migration of HaCaT cells.

**Results:**

The lncRNA XIST and epidermal growth factor receptor (EGFR) were downregulated, while microRNA-126-3p (miR-126-3p) was increased in diabetic ulcer rat skin tissues and high glucose-induced HaCaT cells. In addition, we found that the lncRNA XIST binds to miR-126-3p and that EGFR is directly targeted by miR‑126‑3p. Silencing XIST contributed to upregulated miR-126-3p expression, thus lowering EGFR levels and inhibiting the proliferative and migratory abilities of high glucose-treated HaCaT cells; however, the miR-126-3p inhibitor and overexpression of EGFR reversed this effect.

**Conclusion:**

Decreased lncRNA XIST expression inhibits the proliferative and migratory abilities of high glucose-induced HaCaT cells by modulating the miR-126-3p/EGFR axis, causing delayed wound healing.

**Supplementary Information:**

The online version contains supplementary material available at 10.1186/s13098-024-01260-9.

## Introduction

Diabetes mellitus (DM) is a severe chronic metabolic disorder and ranks as the sixth leading contributor to death in North America [1]. The International Diabetes Federation estimates that the number of individuals with diabetes is expected to increase to 642 million by 2040 [2]. Diabetic foot ulcers (DFUs) are among the most severe diabetic complications, with 15–25% of individuals with diabetes at risk of foot ulcers and 65% of DFUs recurring within 5 years. The lifetime incidence of lower limb amputation is 20%, and the 5-year mortality rate is 50–70%. In 2017, the direct cost of diabetes in the United States was $237 billion, one-third of which was attributed to diabetic foot disease [3–5]. Owing to chronic hyperglycaemic effects, patients with diabetes are prone to neurological and vascular lesions of the lower limbs, which lead to delayed wound healing following injury occurrence [6]. Research has shown that the process of wound healing consists of a series of distinct but partially overlapping phases, including inflammation, proliferation, and remodelling. Keratinocytes, which make up the vast majority of the epidermis, are critically involved in the skin repair process [7]. Following injury, keratinocytes migrate from the wound margins to re-epithelialize damaged tissue and restore the epidermal barrier. Additionally, nonhealing diabetic wounds fall into a state of chronic inflammation that may cause keratinocyte damage, dysfunction, and eventually apoptosis [8]. Previous studies have shown that daily topical treatment of total excisional wounds in C57BL/6 mice with recombinant murine oncostatin M (OSM) improves wound re-epithelialization and accelerates wound closure by activating the JAK/STAT pathway to promote keratinocyte proliferation and migration [9]. There are many new treatments for DFUs [10, 11]; however, the results are not particularly significant. Therefore, it is important to further examine the pathogenesis of this condition and identify new treatment methods.

Long noncoding RNAs (lncRNAs) are transcripts that are longer than 200 nt and do not have notable protein-coding capacity. These molecules exert their influence on the expression of genes in a variety of ways at the epigenetic, chromatin remodelling, transcriptional, and translational levels. Previous studies have shown that lncRNAs H19, URID, MALAT1, and Gas5 are involved in the pathogenic process behind diabetes and associated complications [12–14]. LncRNA X-inactive specific transcript (lncRNA XIST) is one of the lncRNAs that has been extensively studied. This molecule is a set of 15,000–20,000 nt sequences localized in the X chromosome inactivation centre of chromosome Xq13.2 [15]. Serum lncRNA XIST levels have been reported to be low in patients with type 2 diabetic peripheral neuropathy (DPN), and the clinical detection of serum levels may guide the diagnosis and treatment of type 2 DPN [16]. However, the role of XIST in the development of DFUs and related mechanisms have not been reported.

In addition to its initial function in X chromosome dose compensation, the lncRNA XIST acts as a competing endogenous RNA (ceRNA) that contributes to the development of cancers and other human diseases. The ceRNA hypothesis suggests that specific RNAs can be chelated to impair microRNA (miRNA) activity, thereby upregulating target gene expression [13].

MicroRNAs (miRNAs) are short RNAs (20–22 nucleotides) that do not code for proteins and have been recognized over the past decade as important gene expression regulators [13]. Nie et al. discovered a significant reduction in the miRNA-497 expression level in the dermis of diabetic mice, and miRNA-497 intradermal injection around whole dermal wounds in diabetic mice decreased the production of representative proinflammatory cytokines, such as tumour necrosis factor-α (TNF-α), interleukin-6 (IL-6), and interleukin-1β (IL-1β), and effectively accelerated wound closure [17]. The miR-126-3p expression level in the peripheral blood was remarkably elevated in our previous study when comparing patients with type 2 diabetes to healthy individuals, illustrating that miR-126-3p contributes to the onset and progression of diabetes and associated complications [18].

Epidermal growth factor receptor (EGFR) is a protein tyrosine kinase receptor that is expressed on most skin cells. The binding of EGF to EGFR can activate the PI3K/AKT/mTOR signalling pathway [19, 20]. EGFR signalling regulates several cellular functions and performs an integral function in the wound healing response by inducing migration, proliferation, and angiogenesis of fibroblasts, endothelial cells, and keratinocytes [21, 22]. Previous studies by our research team have also demonstrated that topical EGF can effectively promote wound recovery in patients with DFUs [23].

In summary, we propose the following scientific hypothesis of this study: under the pathological conditions of DFUs, the high-glucose environment can induce the downregulation of XIST expression in keratinocytes, thereby resulting in upregulated miR-126-3p expression, which may slow down the proliferation and migration of HaCaT cells through the targeted suppression of the EGFR signalling pathway, thereby resulting in delayed wound healing.

## Materials and methods

### Animal experiment

The SJA Laboratory Animal Co., Ltd. (Hunan, China), supplied the specific pathogen-free (SPF)-grade Sprague Dawley (SD) rats (male, aged 4–6 weeks, and 180–200 g in weight). The rats were housed in a standard 20-22 °C environment with a light/dark cycle of 12 h and an unrestricted supply of food and water. Rats with aberrant body weight and blood glucose levels were screened out after adaptive feeding for one week. The diabetic ulcer and normal groups of rats were established at random; the normal group was administered a standard diet and intraperitoneal sodium citrate buffer (0.1 mol/L, pH 4.5) (Solarbio, Beijing, China); the diabetic ulcer group was administered a high-sugar and high-fat diet for one month and then intraperitoneal injection of 1% streptozotocin (STZ) (40 mg/kg) (Solarbio) dissolved in precooled sodium citrate buffer; a randomized blood glucose level of ≥ 16.7 mmol/L 3 days in a row was considered successful. Those with low blood glucose levels continued to be supplemented with STZ. Eight weeks later, the rats were anaesthetized with isoflurane (RWD, Shenzhen, China), the hair on the back of the rats was removed using an electric shaver, and hair removal cream was applied; then, after 5 min, the rats were scrubbed clean using gauze, the skin surface was disinfected with 75% alcohol, and a circular wound with a diameter of 1 cm was created using a skin biopsy device. This was followed by the application of 50% glacial acetic acid (Xilong Scientific, Guangdong, China) for one consecutive week to simulate an ulcer. The rats were sacrificed, and the skin at the edge of the ulcer was taken for subsequent experiments.

### Histological analysis

After the rat skin tissues were removed, the samples were placed in 4% paraformaldehyde (PFA) (Biochem, Shenzhen, China) for fixation, paraffin-embedded, and cut into 4-μm-thick sections. Subsequently, HE, Masson, and IHC staining experiments were performed. The results were interpreted using a Pathology Slice Scanner (Sunny, Ningbo, China).

### Cell culture and transfection

Zhong Qiao Xin Zhou Biotechnology Co., Ltd. (Shanghai, China), supplied the HaCaT cells, which were incubated at 37 °C in Dulbecco’s modified Eagle’s medium (DMEM, 4.5 g/L) (Gibco, MA, USA) with 10% foetal bovine serum (FBS) (Gibcoat a CO_2_ concentration of 5%. HaCaT cells were incubated in high-glucose medium (27 g/L) for 6 days to simulate a high-glucose environment. LV3-sh-XIST (GenePharma, Shanghai, China) and LV3-oe-EGFR lentivirus (Genechem, Shanghai, China) were used to transfect HaCaT cells. Puromycin (Biosharp, Hefei, China) killed unsuccessfully infected cells and established a stably transfected cell line. A miR-126-3p inhibitor (GenePharma) was employed to transfect cells with Lipofectamine 3000 (Invitrogen, MA, USA), and subsequent experiments were performed after 48 h.

### CCK-8 assay

A 96-well plate was used to seed 1 × 10^4^ cells/well for 3 days. Then, 10 μL of CCK-8 solution (GLPBIO, CA, USA) was added to each well. In a microplate reader (Invitrogen), the absorbance was measured after 2 h at 450 nm.

### Transwell migration assay

Trypsin-digested cells were resuspended in serum-free medium at a density of 1 × 10^6^/mL, and 400 μL of cell suspension was added to a 24-well format cell culture insert (Corning, NY, USA). Subsequently, 1 mL of medium with 20% serum was added to the lower chamber, the cells were rinsed with PBS after 4 days, fixed for 15 min with 4% PFA, and stained with crystal violet solution (Servicebio, Wuhan, China) for 10 min. Thereafter, cotton swabs were used to gently wipe off the cells in the upper chamber, and we placed the samples under a microscope for observation (Zeiss, Germany).

### Wound healing

Five straight horizontal lines were drawn on the back of the six-well plate (Corning) using a marker pen. When the cell density reached 90%, a vertical line was drawn using the tip of a 200-μL tip and then washed with PBS three times to remove cellular debris, and pictures were taken. Next, medium containing 1% FBS was added to continue incubation for 48 h, and photos were taken and recorded at the same position.

### RT-qPCR analysis

Transzol UP (TRANS, Beijing, China)was added to skin tissues and cells to extract RNA, and a Nanodrop system was used to assess quality and concentration. Subsequently, two-step reverse transcription was performed to convert RNA into cDNA using the SweScript RT II First Strand cDNA Synthesis Kit (with gDNA Remover) (Servicebio). RT‒qPCR was conducted on a Step One Plus Real-Time PCR system (Applied Biosystems) utilizing Universal Blue SYBR Green qPCR Master Mix (Servicebio). The internal control was β-actin. For miR-126-3p, reverse transcription was performed using the miRNA 1st Strand cDNA Synthesis Kit (by stem‒loop) (Vazyme, Jiangshu, China), and qPCR was performed using the miRNA Universal SYBR qPCR Master Mix kit (Vazyme). U6 was used as an endogenous control. The 2^−ΔΔCt^ method was employed to determine the relative levels of gene expression. The specific primers are shown in Table 1.

**Table 1:** Primers of Genes

Genes	Primer sequence
Hsa XIST	Forward: GAC ACA AGG CCA ACG ACC TA
	Reverse: TCG CTT GGG TCC TCT ATC CA
Rat XIST	Forward: GCT GGA GAG TGC TGG TTG AC
	Reverse: TGA AGG GAA GAG CTG CTG GA
Has EGFR:	Forward: GGCGTCCGCAAGTGTAAGAA
	Reverse: AGATCGCCACTGATGGAGGT
Rat EGFR	Forward: GCC ACA TCT CCC AAA GCC AA
	Reverse: AGG AGG CAA CCA TAG GGC AT
Hsa β-Actin	Forward: TCTCCCAAGTCCACACAGG
	Reverse: GGCACGAAGGCTCATCA
Rat β-Actin	Forward: AGCCATGTACGTAGCCATCC
	Reverse: ACCCTCATAGATGGGCACAG
Has miR-126-3P	Forward: AACAGTGTCGTACCGTGAGTAATA
	Reverse: GTCGTATCCAGTGCAGGGT
	RT:GTCGTATCCAGTGCAGGGTCCGAGGTATTCGCACTGGATACGACCGCATT
Rat miR-126-3P	Forward: CGC GTC GTA CCG TGA GTA AT
	Reverse: AGT GCA GGG TCC GAG GTA TT
	RT: GTC GTA TCC AGT GCA GGG TCC GAG GTA TTC GCA CTG GAT ACG ACC GCA TT
Hsa U6	Forward: CTCGCTTCGGCAGCACA
	Reverse: AACGCTTCACGAATTTGCGT
	RT:GTCGTATCCAGTGCAGGGTCCGAGGTATTCGCACTGGATACGACAAAATATG
Rat U6	Forward: CCT GCT TCG GCA GCA CA
	Reverse: AAC GCT TCA CGA ATT TGC GT
	RT:GTC GTA TCC AGT GCA GGG TCC GAG GTA TTC GCACTG GAT ACG ACA AAA ATA TG

### Western blot analysis

Radioimmunoprecipitation assay (RIPA) high-efficiency lysis buffer ( Solarbio) mixed with protease inhibitor and phosphatase inhibitor was used to extract proteins from skin tissues and cells. Then, bicinchoninic acid (BCA) )(BioSharp) was employed to measure the protein concentration. Subsequently, sodium dodecyl sulphate‒polyacrylamide gel electrophoresis (SDS‒PAGE) was used to isolate the total protein, followed by transferring it onto PVDF membranes (0.45 μm) (Biochem). The next step involved blocking the membrane with a protein-free rapid blocking solution (Servicebio) for 10 min and incubating it at 4 °C overnight with primary antibodies. The dilutions of antibodies are shown in Table 2. Then, Tris-buffered saline with Tween (TBST) (Servicebio) was used to wash the sample thrice for 5 min each time, and the corresponding HRP-conjugated secondary antibodies (TRANS) were added to incubate the samples for 2 h at 37.5 °C. This step was followed by washing with TBST three times for 5 min each time. Enhanced chemiluminescence (ECL) (FOCUS, Shanghai, China) was applied to PVDF membranes, followed by exposure in a Molecular Imager ChemiDoc XRS + Imaging System (Bio-Rad, CA, USA). Finally, grey value analysis was performed employing ImageJ software.

**Table 2:** The dilutions of antibodies

Protein	Art. No	Dilutions
EGFR	Abcam, ab52894	1:1000
β-Actin	Transfer, HC201	1:5000

### Luciferase reporter gene assay

Wild-type (WT) and mutant (Mut) reporter gene plasmids (Promega, WI, USA) were constructed for XIST and EGFR. The experiment had the following four groups: mimic NC + WT, mimic NC + MUT, mimic + WT, and mimic + MUT. Three replicate wells were set up for each group. The culture solution in the 12-well plate was aspirated, and the transfection mixture was added into the 12-well plate drop by drop, subsequently mixed well, and incubated for 6 h in an incubator; the transfection solution was aspirated and discarded, and 500 μL of complete culture medium was added. Incubation was carried out for 24 h at 37 °C, during which time 5% carbon dioxide was introduced. Next, we continued to incubate the samples for 24 h. Afterwards, 100 μL of cell lysate (Promega) was taken and added to the enzyme-labelling plate. Then, 10 μL of firefly luciferase reaction solution was added. The enzyme plate was shaken to mix well, and the activity of firefly luciferase was detected; subsequently, 10 μL of sea kidney luciferase reaction solution was added. The enzyme plate was shaken to mix well, and the sea kidney luciferase activity was assessed.

### Statistics

All experiments were conducted in triplicate. The data are shown as the means ± standard deviations. For identification of significant variations between the two groups, Student’s t tests were used. Multiple groups were compared via one-way ANOVA. GraphPad Prism 9.5 (GraphPad Prism Software, Inc., San Diego, CA) was used to analyse all data. The significance level was established at *P* values of < 0.05.

## Results

### The lncRNAs XIST and EGFR were reduced while miR-126-3p was upregulated in the skin tissues of rats with diabetic ulcers

**Fig. 1:**
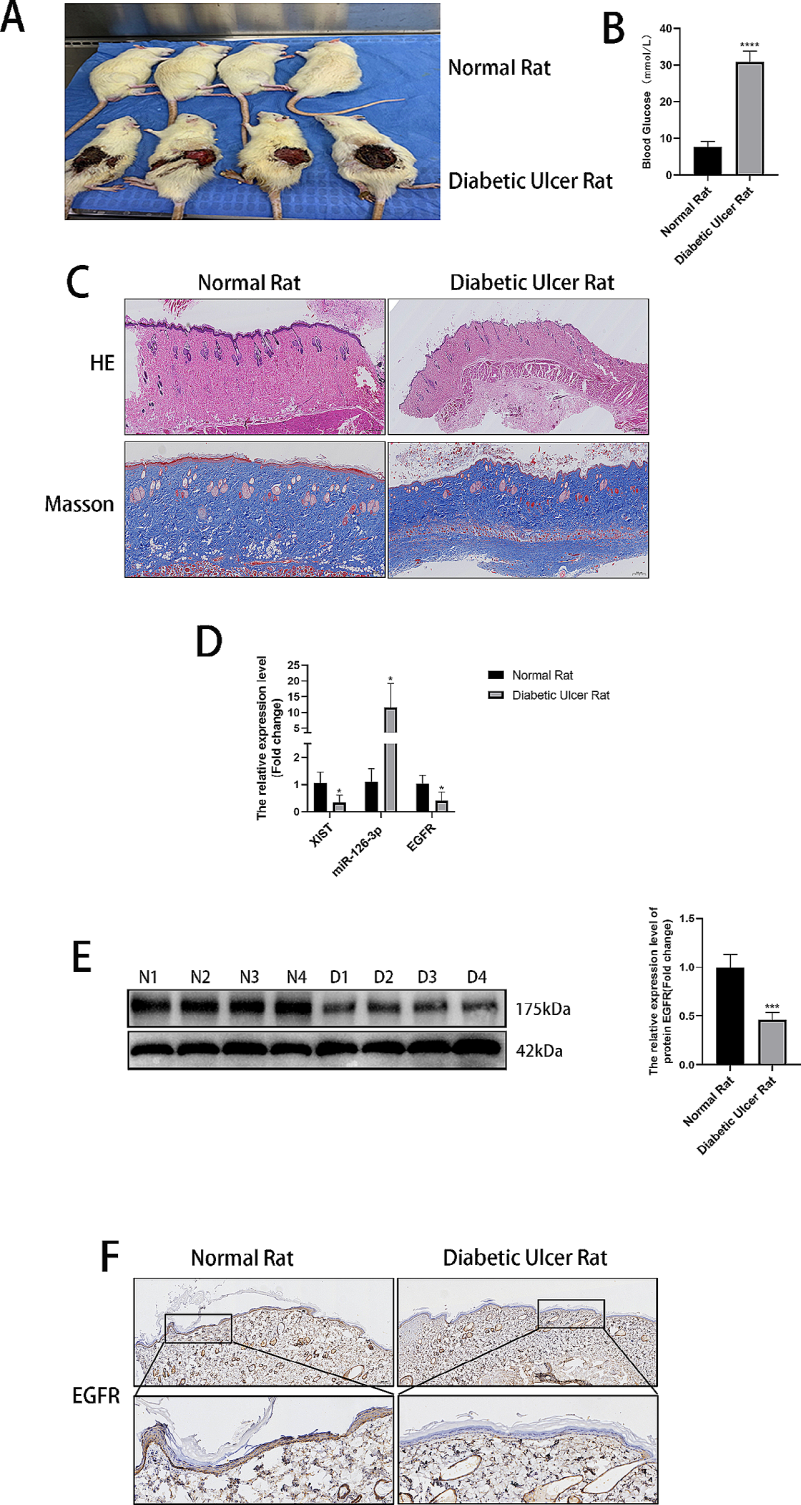
Changes in biochemical indices, pathological features, and gene expression in normal rats and rats with diabetic ulcers. (**A**) Image of the normal and diabetic rat groups. (**B**) Glucometer measurement of random blood glucose levels in the tail vein. (**C**) HE staining of the normal and diabetic ulcer groups (magnification: 20×; scale bar = 500 μm). Masson staining of skin tissues (magnification: 50×; scale bar = 200 μm). (**D**) The relative expression of lncRNA XIST, miR-126-3p, and EGFR determined via RT-qPCR. (**E**) The relative expression of EGFR determined via Western blots. (**F**) The expression of EGFR in skin tissues was assessed using IHC (magnification: 50×; scale bar = 200 μm) (magnification: 200×; scale bar = 50 μm). *P < 0.05, ***P < 0.001, ****P < 0.0001

First, to explore the differences in miR-126-3p, lncRNA XIST, and EGFR expression in rat skin tissues, we established a diabetic rat ulcer model, as shown in Fig. 1A. The blood glucose level of the diabetic ulcer group was substantially elevated relative to that of the normal group, as shown in Fig. 1B. Moreover, HE and Masson staining results showed that the skin structure of the normal rats had clear layers, the epidermis was uniformly thick and thin, the surface was mildly keratinized, the granular layer existed, and the structure of skin appendages in the dermis was normal. However, the skin structure of the rats with diabetic ulcers had slightly fuzzy layers, the epidermis was thinned, the granular layer disappeared, the structure of skin appendages and blood vessels was reduced, and collagen was reduced in the dermis. Skin appendages and blood vessels were reduced, and collagen fibres were reduced, as depicted in Fig. 1C. Furthermore, RT‒qPCR, Western blot and IHC results showed that in comparison to the normal rats, the rats with diabetic ulcers had decreased XIST and EGFR expression in the skin tissues, whereas the miR-126-3p content increased (Fig. 1D-F).

### Suppression of cell proliferation and migration under high-glucose conditions

**Fig. 2:**
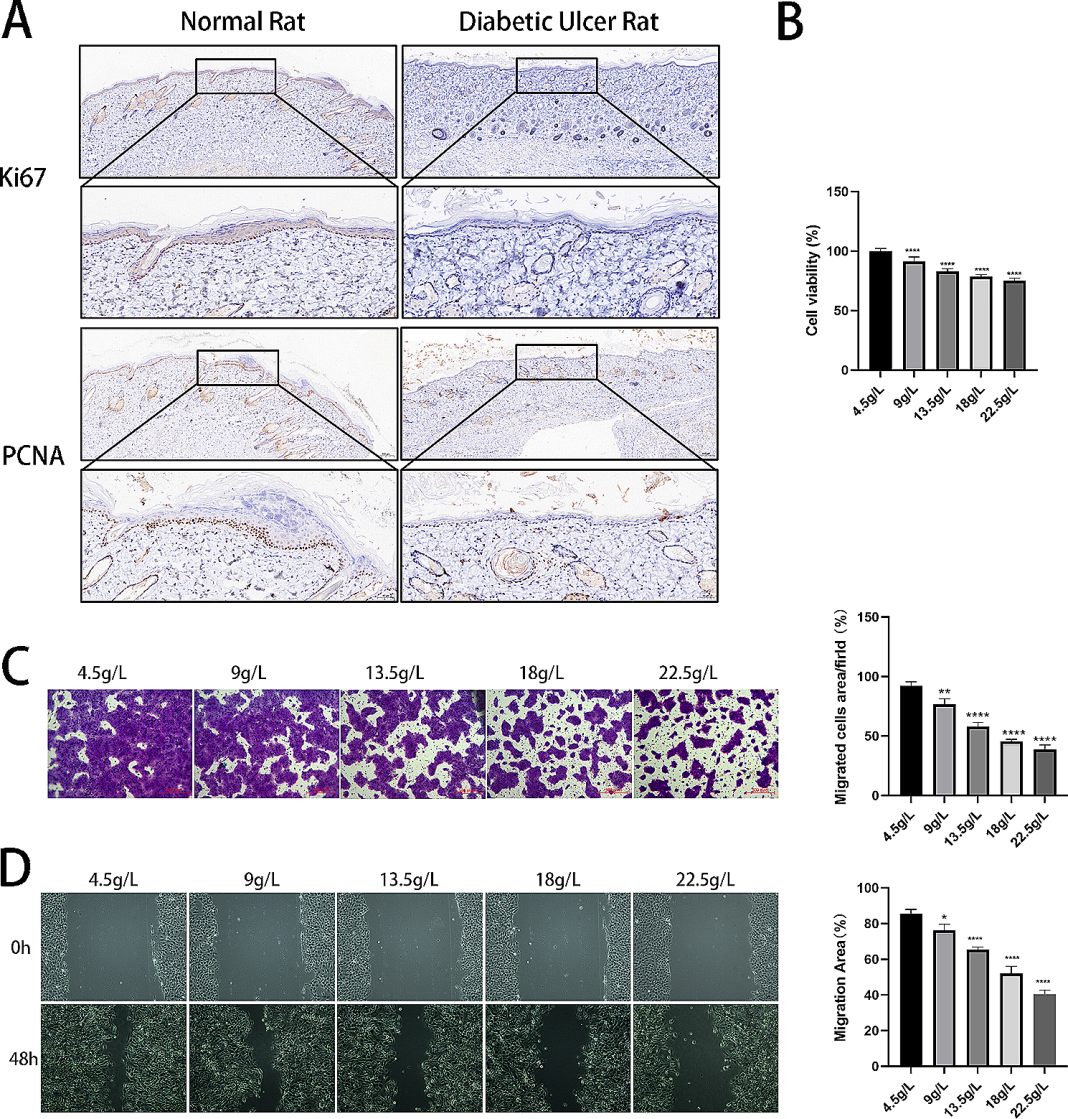
Suppression of cell proliferation and migration in the high-glucose cell model. (**A**) The relative expression of Ki67 and PCNA as measured using IHC. (**B**) The CCK-8 test results for the viability of the cells. (**C**) Transwell assay-calculated area for cell migration. (**D**) The cell migration distance was measured by wound healing assays at 0 and 48 h. *P < 0.05, **P < 0.01, ****P < 0.0001, *VS* 4.5 g/L

To simulate the cell damage caused by hyperglycaemia in patients with DFUs, we developed an in vitro hyperglycaemia model of HaCaT cells. The effectiveness of the model was verified by testing the cell viability and migration. As shown in Fig. 2A, the value-added indicators Ki67 and PCNA in the skin of rats with diabetic ulcers were decreased compared with those in normal skin. A viability analysis of cells was performed utilizing the CCK-8 assay. After the cells were treated with different high-glucose concentrations, the relative cell activity gradually decreased, as shown in Fig. 2B. Cell scratch and Transwell assays were performed to determine cell migratory capacity, and the number of migrated cells decreased, as shown in Fig. 2C-D. Finally, 4.5 g/L was selected for the normal glucose (NG) group, and 22.5 g/L was selected for the high glucose (HG) group.

### Silencing XIST inhibits the proliferative and migratory capacities of HaCaT cells treated with high glucose

**Fig. 3:**
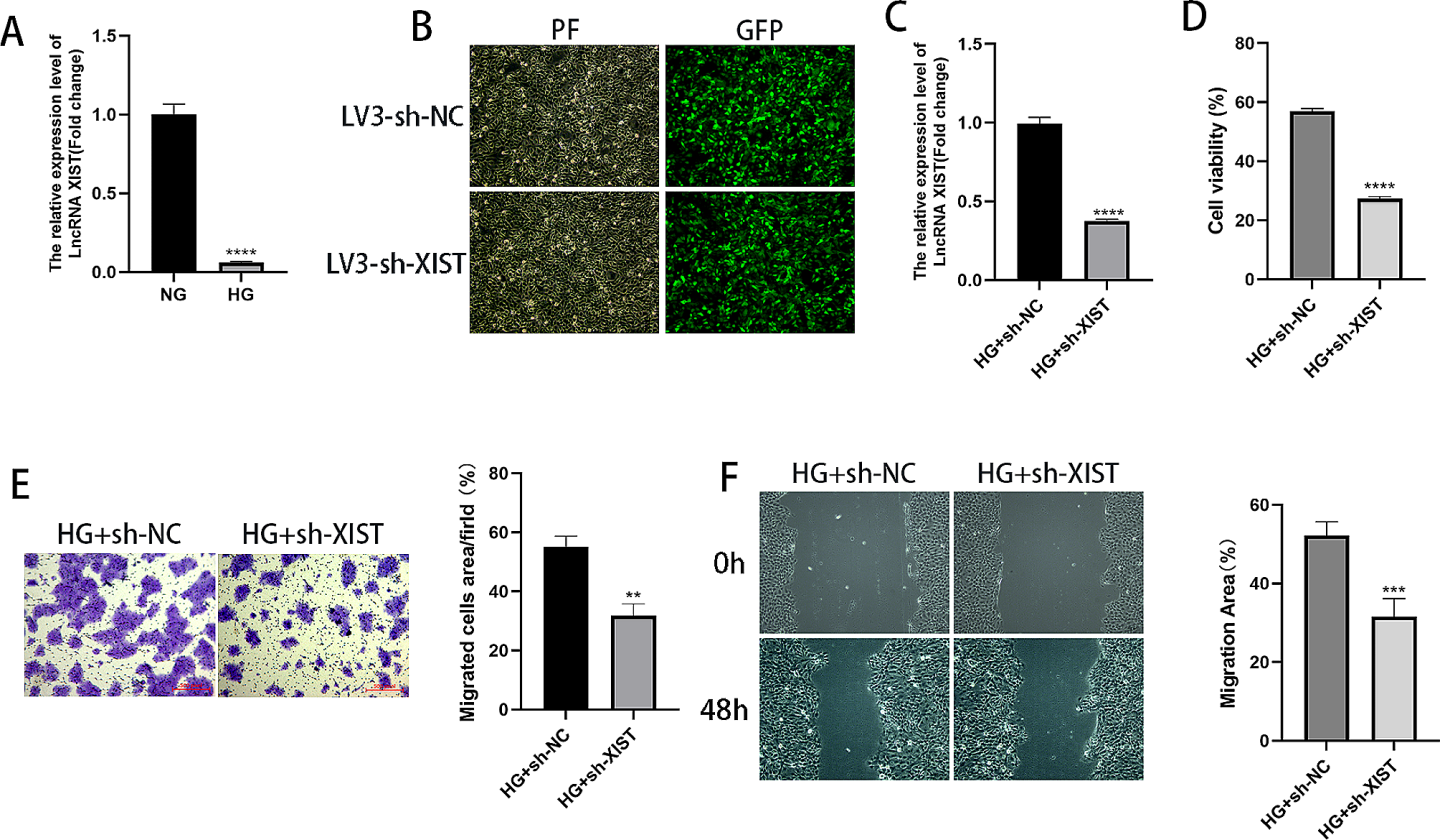
Silencing XIST inhibits the proliferative and migratory capacities of HaCaT cells treated with high glucose. (**A**) The relative concentrations of lncRNA XIST as observed using RT-qPCR. (**B**) Inverted fluorescence microscopy was used to observe the cell lentiviral transfection efficiency. (**C**) The relative expression levels of the lncRNA XIST measured using RT-qPCR. (**D**) Cell proliferative rate as measured using CCK-8 assays. (**E**) Migrated cells were counted and analysed using Transwell assays. (**F**) Wound healing assays and images taken at 0 and 48 h. **P < 0.01, ***P < 0.001, ****P < 0.0001

Under constant high-glucose stimulation, intracellular genes were dysregulated. XIST was expressed at low levels in HG-cultured HaCaT cells, as depicted in Fig. 3A. To examine the impact of XIST on DFUs in vitro, we aimed to generate a lentivirus of oe-XIST, but no genetic company could perform generate this construct. The expression of XIST was knocked down by transfection with sh-XIST in HaCaT cells. First, the LV3-sh-XIST lentiviral vector was constructed and used to infect HaCaT cells. As shown in Fig. 3B, a large amount of green fluorescence was visible under the inverted fluorescence microscope, indicating that the lentivirus was transfected into the cells with high transfection efficiency; RT-qPCR assays again illustrated that the sh-XIST lentivirus decreased the XIST expression in the cells, as shown in Fig. 3C. The cell proliferative capacity of the cells was evaluated using the CCK-8 test; we found that compared to that of the NG group, the cell viability of the HG group was substantially decreased; simultaneously, the cell viability of the sh-XIST group was further reduced relative to that of the sh-NC group, as shown in Fig. 3D. Moreover, using cell scratch and Transwell assays, we assessed cell migration. The results from both experiments showed that the HG group had significantly reduced cell migration compared with the NG group, and the sh-XIST group had further reduced cell migration compared with the sh-NC group (Fig. 3E-F). This finding shows that cell proliferation and migration are significantly inhibited under high glucose, and silencing XIST aggravates the inhibitory effect of cell proliferation and migration.

### LncRNA XIST binds to miR-126-3p

**Fig. 4:**
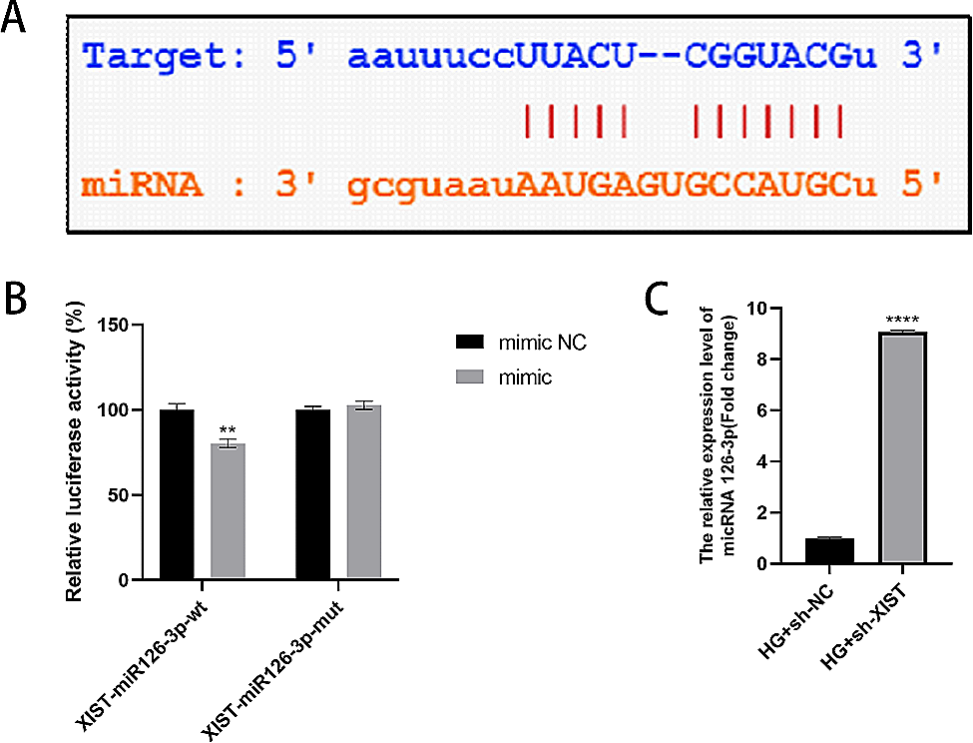
XIST acts as a sponge for miR-126-3p. (**A**) The StarBase online bioinformatics database predicts the potential binding site of XIST and miR-126-3p. (**B**) Relative luciferase activity determined using the dual luciferase reporter gene method. (**C**) The relative expression of miR-126-3p determined using RT-qPCR. **P < 0.01, ****P < 0.0001

The existence of complementary sequences between lncRNA XIST and miR-126-3p was predicted using the StarBase online bioinformatics database to better explore the interaction, as depicted in Fig. 4A. Furthermore, this hypothesis was validated using a dual luciferase reporter gene assay, which showed the suppression of the luciferase activity of lncRNA XIST-miR-126-3p-Wt by the miR-126-3p mimic, demonstrating that lncRNA XIST can compete for binding with miR-126-3p, as shown in Fig. 4B. As expected, the sh-XIST group had substantially upregulated miR-126-3p expression, as shown in Fig. 4C.

### A mir-126-3p inhibitor enhances the proliferation and migration of HaCaT cells triggered by high glucose

**Fig. 5:**
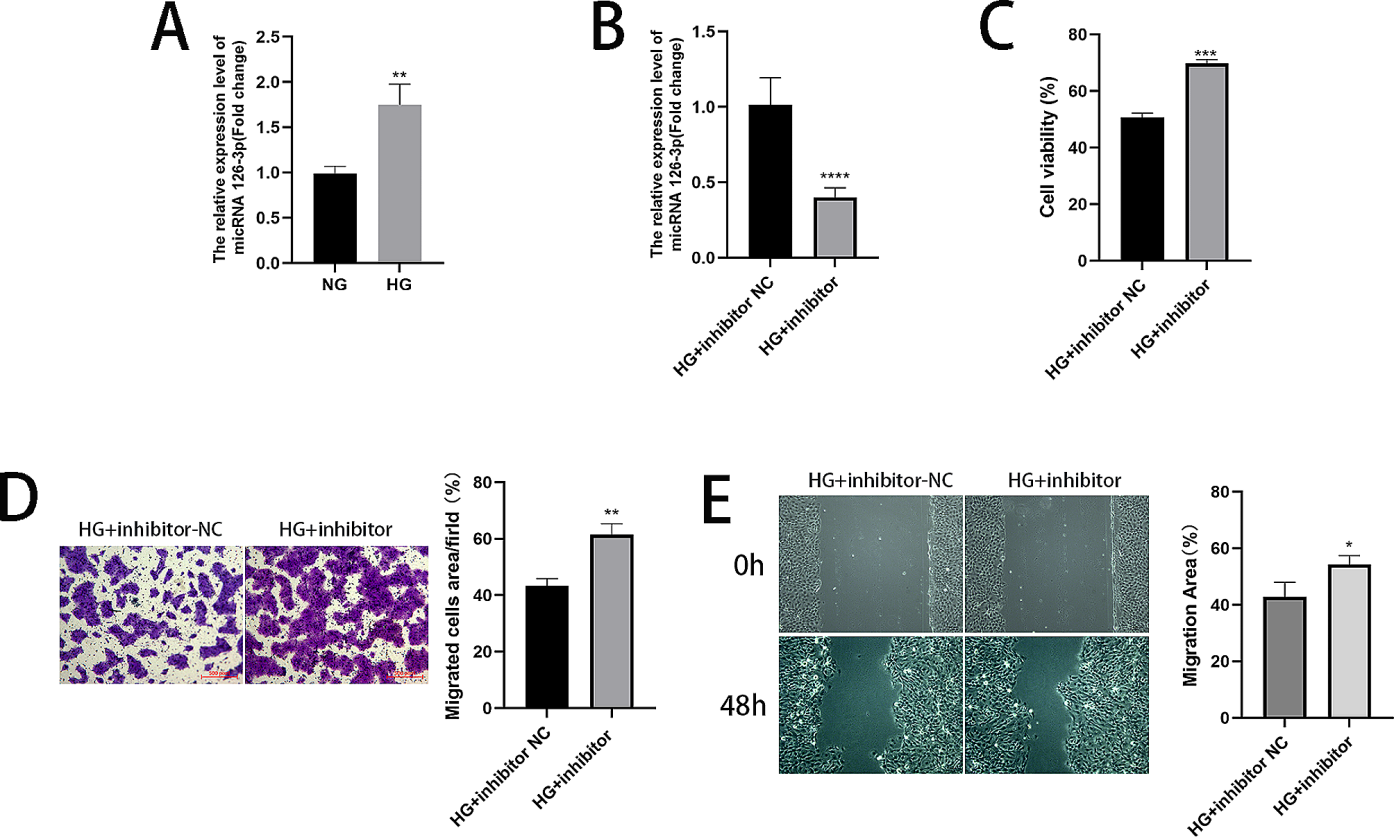
A miR-126-3p inhibitor enhances the proliferation and migration of HaCaT cells triggered by high glucose. (**A**-**B**) The relative miR-126-3p expression levels were determined using RT-qPCR. (**C**) Cell proliferative ability as measured using CCK-8 assays. (**D**) Migrated cells were counted and analysed using Transwell assays. (**E**) Wound healing assays and images taken at 0 and 48 h. *P < 0.05, **P < 0.01, ***P < 0.001, ****P < 0.0001

As demonstrated by RT-qPCR, elevated expression levels of miR-126-3p were found in the HG group in comparison to the NG group, as shown in Fig. 5A. Furthermore, for determination of the involvement of miR-126-3p in DFUs, a miR-126-3p inhibitor fragment was first constructed and transfected into HaCaT cells by Lip3000. RT-qPCR analysis demonstrated the downregulation of miR‑126-3p expression in HaCaT cells following transfection with the miR-126-3p inhibitor, as shown in Fig. 5B. Additionally, CCK-8, wound healing, and Transwell assays showed that the miR-126-3p inhibitor enhanced the cell proliferation and migration (Fig. 5C-E). This finding demonstrated that the miR-126-3p inhibitor was able to partially reverse the suppressive impact of high glucose on cell proliferation and migration.

### EGFR is directly targeted by miR‑126‑3p

**Fig. 6:**
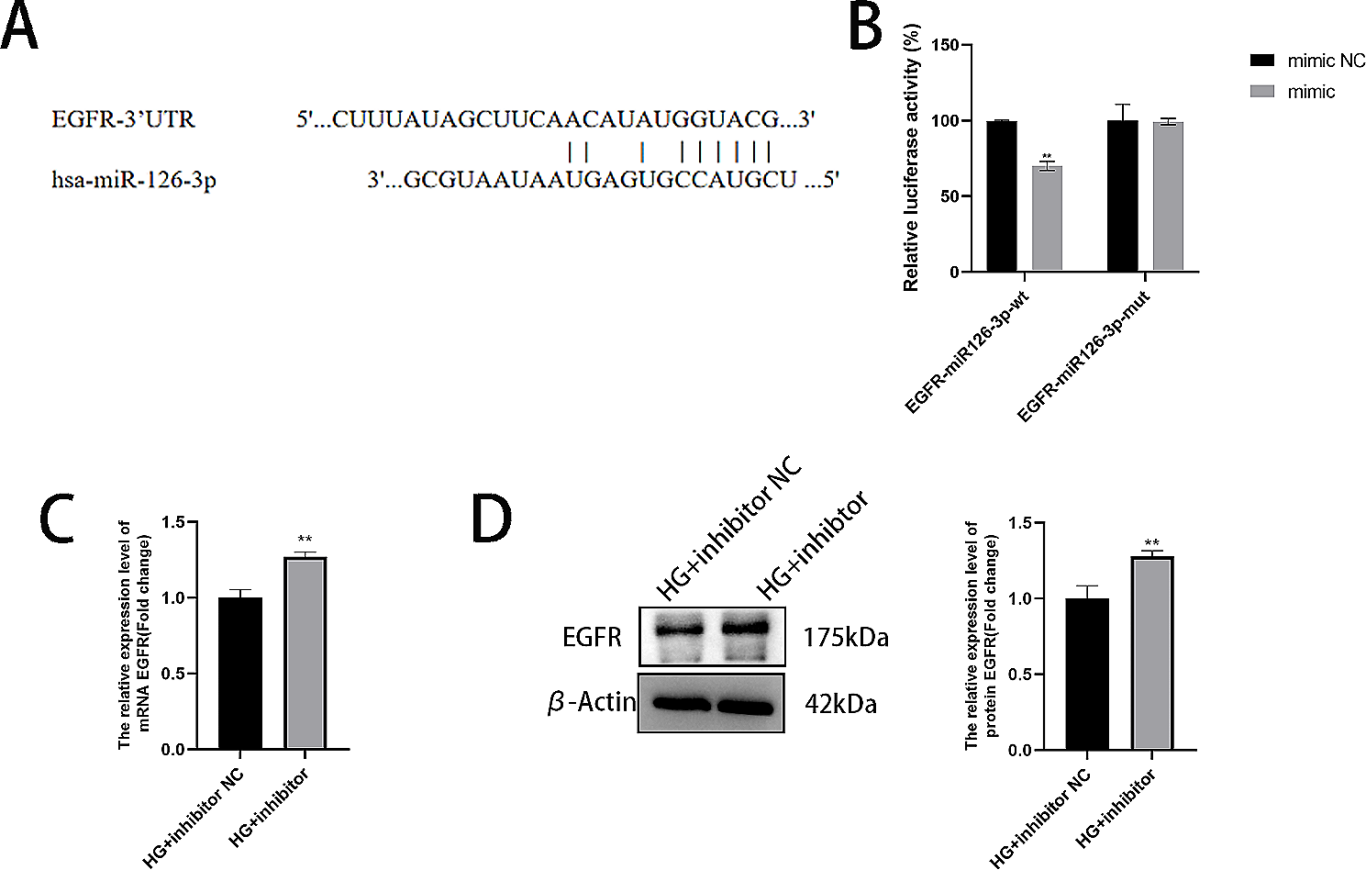
EGFR is directly targeted by miR‑126‑3p. (**A**) The potential EGFR and miR-126-3p binding sites predicted using the online bioinformatics database StarBase. (**B**) Dual luciferase reporter gene assay for measuring luciferase activity. (**C**-**D**) Western blotting and RT-qPCR were performed to determine the relative EGFR expression levels. **P < 0.01

To demonstrate that EGFR is a downstream miR-126-3p target gene, we used a bioinformatics platform to predict the binding sequences of miR-126-3p and EGFR, as depicted in Fig. 6A. EGFR-WT luciferase reporter gene expression was regulated by miR-126-3p, and upon binding site mutation (EGFR-MUT), the reporter gene expression was reversed (Fig. 6B). Furthermore, this finding was verified using RT-qPCR and Western blot experiments. We discovered that the mRNA and protein expression of EGFR in HaCaT cells was increased by the inhibitor miR‑126-3p, as shown in Fig. 6C-D.

### EGFR overexpression enhances the proliferation and migration of HaCaT cells in high-glucose environments

**Fig. 7:**
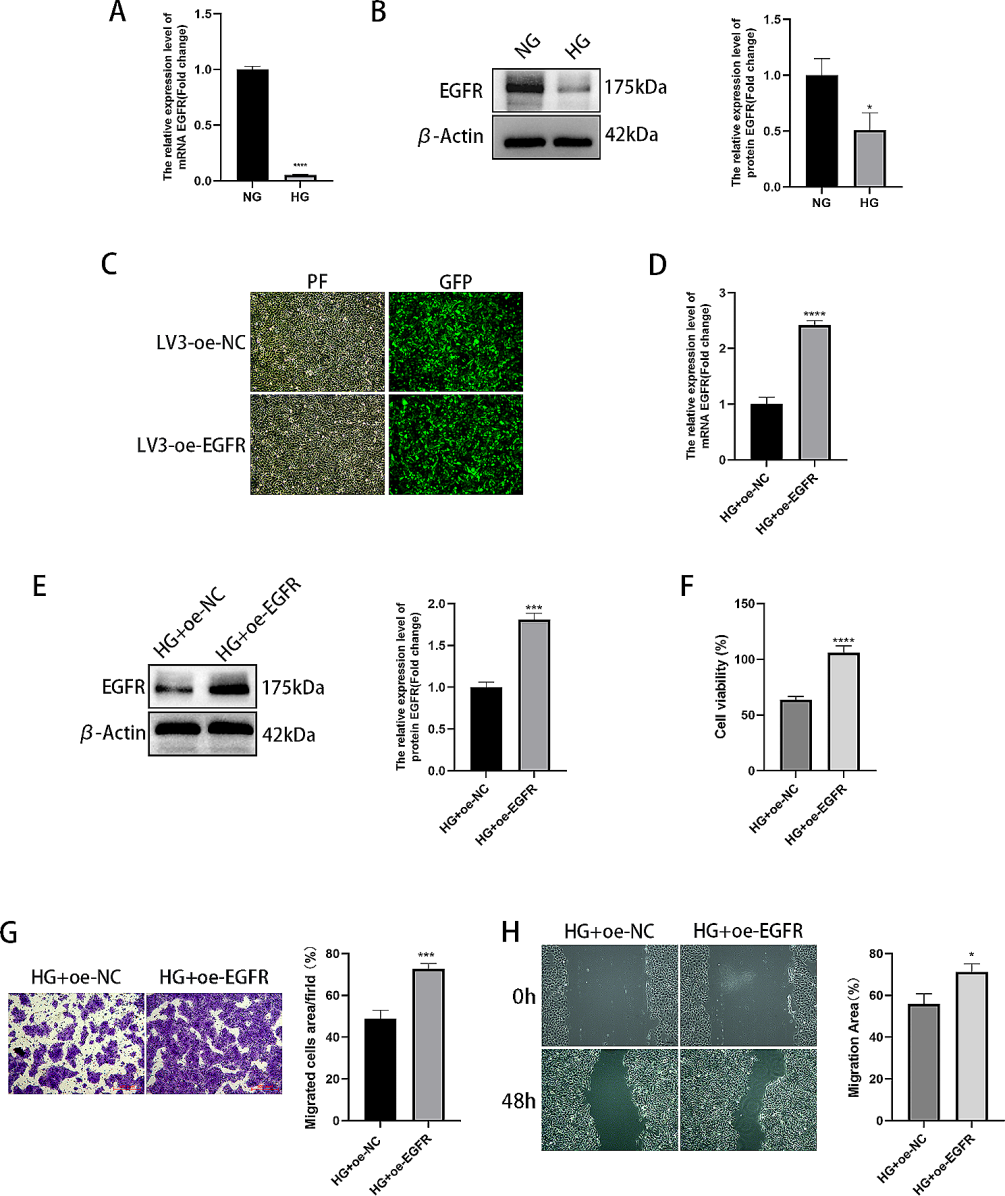
EGFR overexpression promotes the proliferation and migration of HaCaT cells in high-glucose environments. (**A**) RT-qPCR was used to determine the relative EGFR expression levels. (**B**) The relative EGFR expression levels were determined using Western blotting. (**C**) Inverted fluorescence microscopy was used to observe the cell lentiviral transfection efficiency. (**D**) The relative EGFR expression levels were determined using RT-qPCR. (**E**) The relative EGFR expression was estimated using Western blotting. (**F**) Cell proliferative ability measured using CCK-8 assays. (**G**) Migrated cells were counted and analysed using Transwell assays. (**H**) Wound healing assay and images taken at 0 and 48 h.*P < 0.05, ***P < 0.001, ****P < 0.0001

Furthermore, the EGFR level was determined using RT-qPCR and Western blotting. As displayed in Fig. 7A-B, the EGFR content was reduced in cells induced by high glucose. For determination of the impact of EGFR overexpression on the biofunctionality of HaCaT cells in a high-glucose environment, an EGFR overexpression lentivirus was constructed and used to infect HaCaT cells. Lentivirus transfection was observed using an inverted fluorescence microscope, and the results showed that a large amount of green fluorescence was observed under the microscope, indicating that the lentivirus was transfected into the cells, as shown in Fig. 7C. The transfection efficiency was determined using RT-qPCR and Western blotting, as shown in Fig. 7D-E. EGFR was highly expressed following EGFR overexpression. Moreover, EGFR overexpression increased cell proliferation and migration, as illustrated in Fig. 7F-H.

### Rescue

**Fig. 8:**
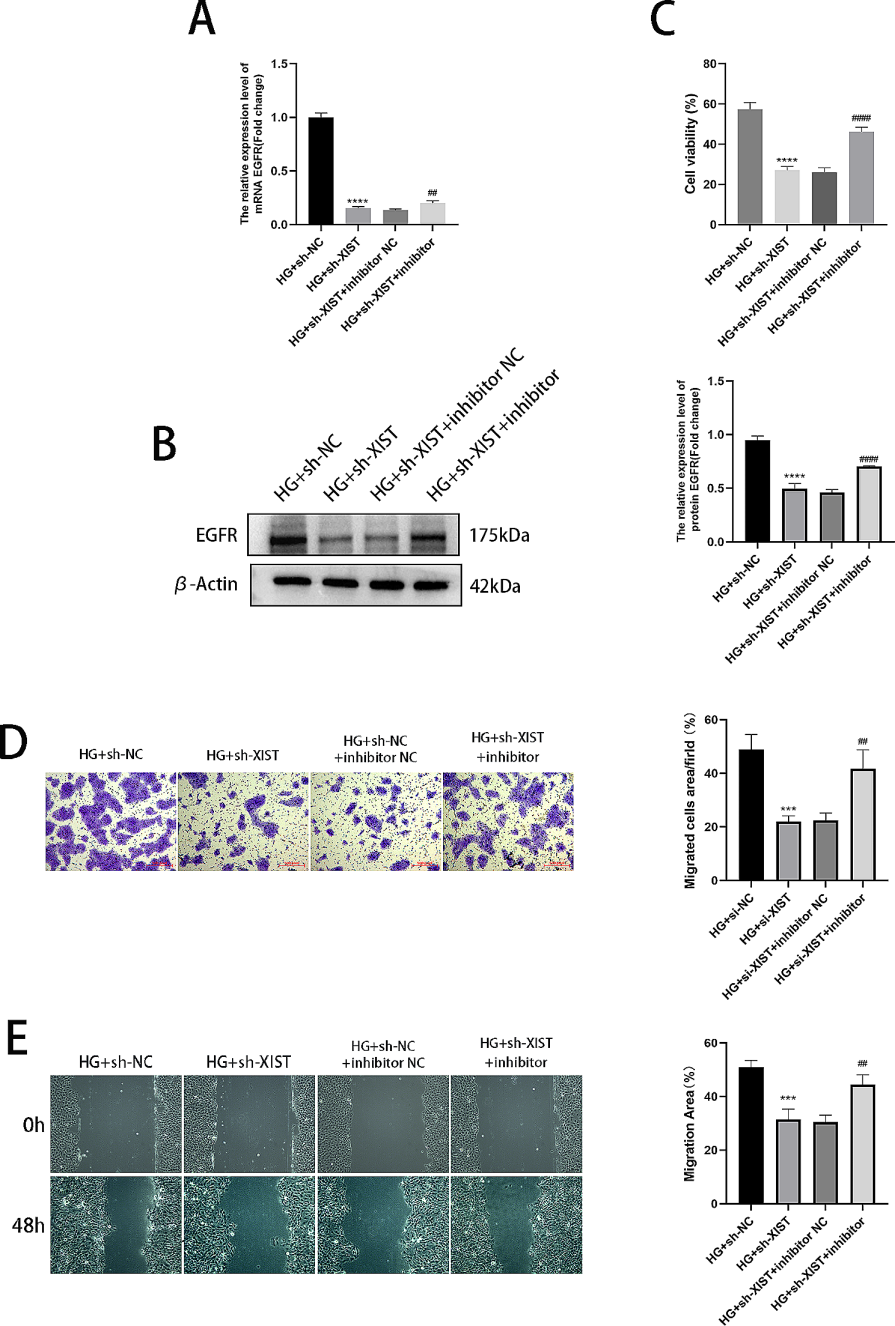
XIST targets miR-126-3p to regulate HG-induced HaCaT cell viability and migration. (**A**) The relative EGFR mRNA expression levels were determined using RT-qPCR. (**B**) The relative EGFR expression levels measured using Western blot. (**C**) Cell proliferation as measured using CCK-8 assays. (**D**) Migrated cells were counted and analysed using Transwell assays. (**E**) Wound healing assays and images taken at 0 and 48 h. ***P < 0.001, ****P < 0.0001, *VS* HG + sh-NC; ##*P* < 0.01, ####*P* < 0.0001, *VS* HG + sh-XIST + inhibitor NC

For further confirmation that the lncRNA XIST regulates the cellular biofunctionality of HaCaT cells in high-glucose environments through the miR-126-3p/EGFR axis, cells were cotransfected with sh-XIST and miR-126-3p inhibitor. As illustrated in Fig. 8A-B, the miR-126-3p inhibitor reversed the decrease in EGFR expression induced by sh-XIST. Conversely, functional experiments showed that silencing XIST inhibited HG-stimulated HaCaT cell proliferation and migration; however, the miR-126-3p inhibitor eliminated these effects, as depicted in Fig. 8C-E.

## Discussion

To date, the mechanisms of hyperglycaemic injury have been studied extensively, achieving remarkable progress in recent years. Increasing evidence suggests that lncRNAs regulate target gene expression levels through transcriptional, epigenomic, or post-transcriptional mechanisms and perform key regulatory roles in critical cellular functions, such as differentiation, proliferation, migration, invasion, and apoptosis [24]. Additionally, lncRNAs detected in body fluids have been recognized as candidate diagnostic, prognostic, and progression-monitoring biological markers, as well as possible new pharmacological targets for treating human diseases [15].

Our previous study reported that miR-126-3p was highly expressed in the peripheral blood of patients with type 2 DM, and topical EGF could effectively promote wound recovery in patients with DFUs. Interestingly, bioinformatics analysis suggested that miR-126-3p had binding sites for XIST and the mRNA of EGFR, a key gene promoting wound repair in the organism. Accordingly, we hypothesized that a competitive endogenous RNA regulatory relationship exists among XIST, miR-126-3p, and EGFR. Therefore, our next experiment focused on the molecular mechanism and biological roles of lncRNA XIST in DFUs.

LncRNA XIST is an important modulator of cell growth and development. In particular, the lncRNA XIST has a remarkable function in the occurrence of several cancer types, including brain tumours, leukaemia, and liver, breast, bladder and lung cancers [15, 25]. Federico Biscetti ‘s research found that rs 3134069, rs 2073617, and rs 2073618 variant genotypes of the OPG gene are significantly and independently associated with the increased risk of ischemic stroke in diabetic patients [26]. Previous studies have shown a negative correlation between low serum lncRNA XIST levels and high miR-30d-5p levels in patients with type 2 DPN. The clinical determination of these two serum levels may have a guiding role in the diagnosis and treatment of type 2 DPN [16]. Liu et al. reported that trigeminal sensory neurons in diabetic mice showed decreased XIST and SIRT1 expression and increased miR-30d-5p expression [27]. Under high-sugar conditions, XIST expression was suppressed; miR-21-5p, miR-30, and miR-30d-5p were elevated; and AVEN, sirtuin1, and BECN-1 were decreased [28–30].

In this study, diabetic rats were established by combining a high-sugar and high-fat diet with intraperitoneal injection of STZ; subsequently, a total skin defect was created on their backs, and a diabetic ulcer model was successfully developed by applying glacial acetic acid. To simulate the high-glucose environment of the cells, we added H-glucose to the original culture medium and selected the appropriate concentration. We observed the downregulation of lncRNA XIST and EGFR and the upregulation of miR-126-3p in diabetic ulcerated rat tissues and the high glucose-induced HaCaT cell injury model.

The ceRNA mechanism is a common mechanism for lncRNAs, which upregulate certain mRNAs by taking up miRNAs. The mesenchymal stem cell-derived exosome lncRNA H19 upregulates PTEN via microRNA-152-3p to promote wound healing in DFUs [31]. Moreover, in fibroblast-like synoviocytes, there was a substantial increase in XIST expression in diabetic tissues and cells compared to healthy controls. Additionally, XIST downregulation inhibited the cell proliferative rate and increased the apoptotic rate. MiR-126-3p was identified as a XIST target gene by analysis of luciferase reporter genes. Additionally, by reducing the expression levels of p-p65 and p-IκBα in RA-FLSs, miR-126-3p overexpression could inhibit the NF-κB signalling pathway [32]. A study by Zhao et al. showed that microRNA-126-5p targets EGFR to inhibit hepatocellular carcinoma cell proliferation, invasion, and migration [33]. To validate the ceRNA regulatory role between XIST/miR-126-3p/EGFR, we used molecular biology techniques. The binding domains were predicted using a bioinformatics database. A dual luciferase reporter gene assay showed that there were binding sites between miR-126-3p and XIST or EGFR. RT-qPCR and Western blot results revealed that following XIST silencing, the miR-126-3p content was increased, and the EGFR content was decreased. The miR-126-3p inhibitor increased EGFR expression.

Upregulation of XIST expression was reported to antagonize high glucose-induced apoptosis and restore migration [29]. Furthermore, XIST protects podocytes against HG-induced damage, induces autophagy in podocytes, and suppresses the progression of diabetic nephropathy by regulating the miR-30d-5p/BECN1 axis [28]. The upregulation of miR-129 and miR-335 in vivo promotes wound closure by inhibiting Sp1-mediated matrix metalloproteinase (MMP)-9 expression, enhancing keratinocyte motility, regaining skin density, and replenishing collagen in a diabetic wound model [34]. Lin reported that inhibiting miR-217 enhanced the inflammatory response, increased angiogenesis, and hastened wound healing in rats with DFUs by enhancing the HIF-1α/VEGF pathway [35]. In this experiment, we observed that cell proliferation and migration were inhibited in high-glucose environments. Additionally, XIST silencing further aggravated the inhibitory effect on HaCaT cells treated with high glucose, but the miR-126-3p inhibitor partially reversed this effect.

Defects in keratinocyte migration and proliferation frequently lead to defective wound repair [36]. Hyperglycaemia has been found to disrupt the protein synthesis, migration, and proliferation of keratin-forming cells and fibroblasts and is also a potential cause of endothelial cell dysfunction [24]. Keratinocyte state transition is regulated by different wound microenvironmental factors, including MMPs, chemokines, cytokines, and growth factors [7]. EGFRs play a central role in several aspects of keratinocyte biology. In the normal epidermis, EGFRs are essential for the autocrine growth of this renewed tissue, thereby inhibiting terminal differentiation, promoting cell survival, and regulating cell migration during epidermal morphogenesis and wound healing. In keratinocyte carcinoma, EGFR signalling inhibition is a potent adjuvant for cancer treatment [21, 37, 38]. Xu et al. showed that high glucose may impair the EGFR/PI3K/AKT pathway via reactive oxygen species (ROS), thereby resulting in delayed corneal epithelial wound healing [39]. This finding aligns with the outcomes of our experiments. Furthermore, we observed a reduction in the expression level of EGFR in the sh-XIST group relative to the sh-NC group. In conclusion, after binding to its ligand EGF, EGFR can modulate the expression of downstream genes, thereby affecting cell survival, proliferation, migration, and inflammation.

## Conclusion

**Fig. 9:**
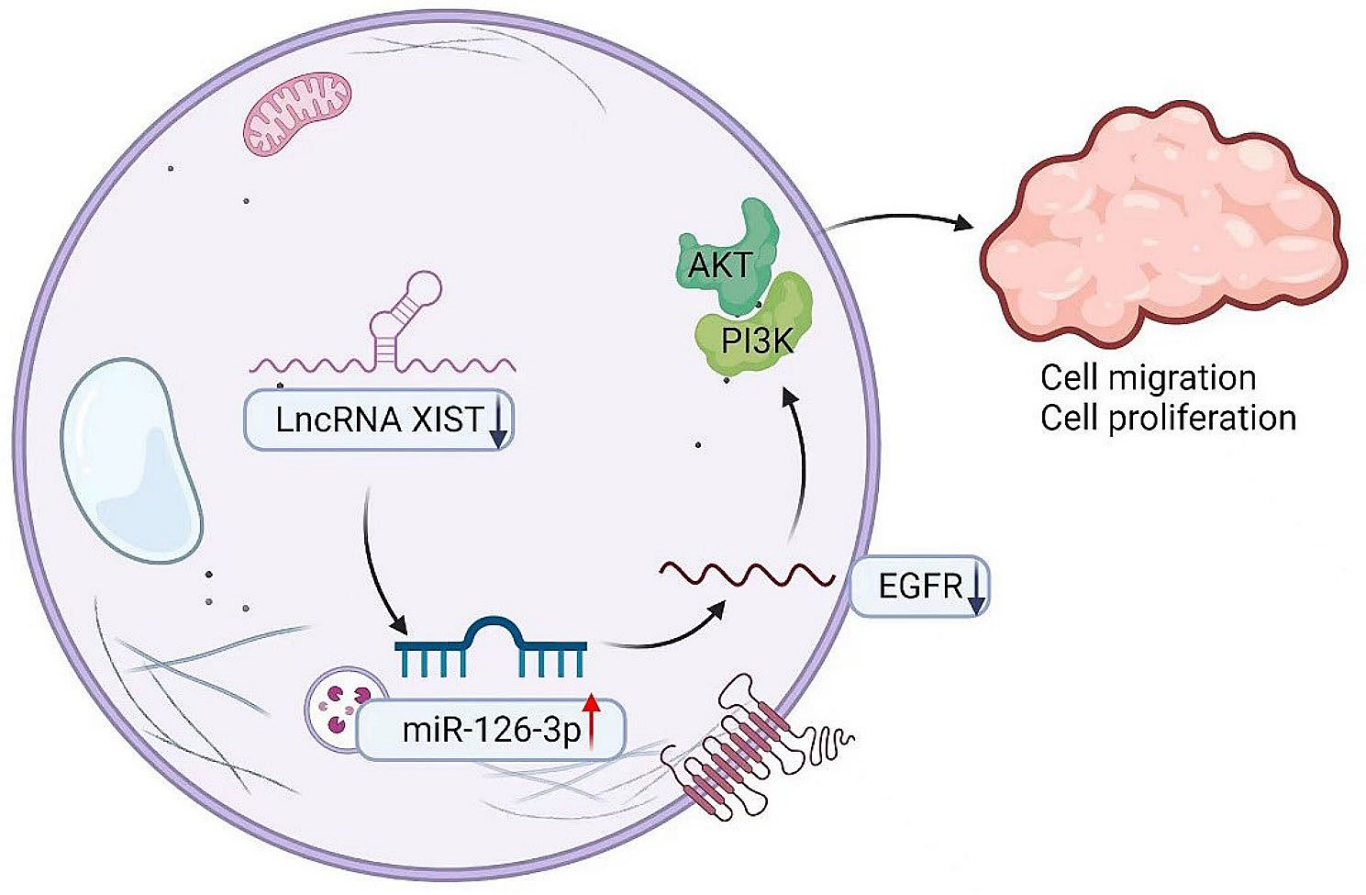
Schematic representation of the lncRNA/miR-126-3p/EGFR axis

Our study reveals the mechanism by which the lncRNA XIST regulates high-glucose injury in HaCaT cells by targeting the miR-126-3p/EGFR axis, and XIST silencing could inhibit proliferation and migration in high glucose-treated HaCaT cells, as shown in Fig. 9, thereby demonstrating the significant role of the lncRNA XIST in DFUs. This mechanism will help to elucidate the pathogenesis of DFUs and provide new ideas and approaches for the clinical treatment of DFUs.

### Electronic supplementary material

Below is the link to the electronic supplementary material.

Supplementary Material 1: Western blot

## Data Availability

Data will be made available on request.
