# Supplementary figures and images for: Long noncoding RNA XIST promotes cell proliferation and migration in diabetic foot ulcers through the miR-126-3p/EGFR axis
Source: Diabetol Metab Syndr. 2024 Feb 6;16:35. doi: 10.1186/s13098-024-01260-9 (PMC10845590; doi:10.1186/s13098-024-01260-9)

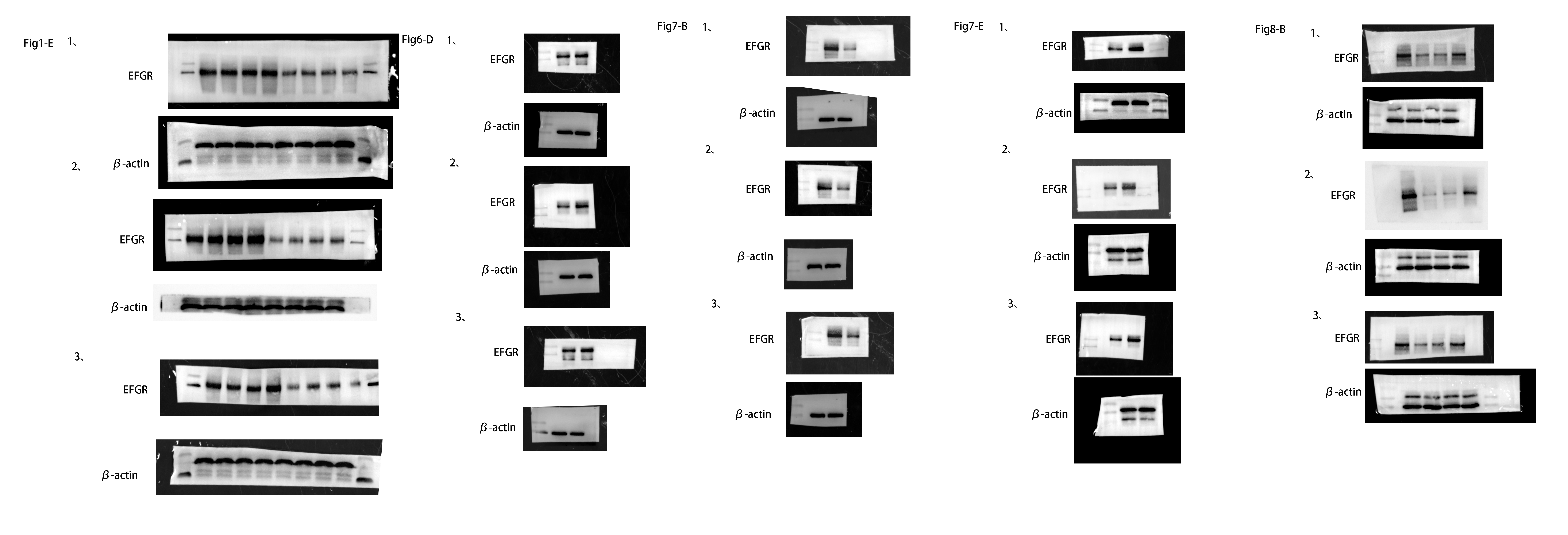

Supplement: Supplementary file 1 — Supplementary Material 1: Western blot [file 13098_2024_1260_MOESM1_ESM.png]
